# Overcoming Chemoresistance in Glioblastoma: Mechanisms, Therapeutic Strategies, and Functional Precision Medicine

**DOI:** 10.3390/ijms27052207

**Published:** 2026-02-26

**Authors:** Maria Y. Kordyukova, Timofey K. Bulgakov, Maria A. Sorokina, Olga M. Kudryashova, Tatiana O. Abakumova, Valeriya I. Meshcheryakova, Ilya V. Grigoriev, Ilya V. Senko, Evgeny K. Shevchenko, Vsevolod V. Belousov

**Affiliations:** 1 Federal Center of Brain Research and Neurotechnologies, Federal Medical and Biological Agency, 117513 Moscow, Russia; bulgakov.t@fccps.ru (T.K.B.);; 2 Shemyakin-Ovchinnikov Institute of Bioorganic Chemistry, Russian Academy of Sciences, 117997 Moscow, Russia; 3 Laboratory of Synthetic Neurotechnologies, Pirogov Russian National, Research Medical University, Ministry of Health of the Russian Federation, 117513 Moscow, Russia; 4 Life Improvement by Future Technologies (LIFT) Center, 121205 Moscow, Russia

**Keywords:** glioblastoma, chemotherapy, personalized medicine, drug screening, therapy resistance, chemoresistance

## Abstract

Glioblastoma (GBM) is the most common primary malignant brain tumor in adults and remains highly lethal, with median overall survival rarely exceeding 15 months despite maximal surgical resection, radiotherapy, and temozolomide-based chemotherapy. Therapeutic resistance in GBM is driven by intrinsic tumor cell adaptations, extensive inter- and intratumoral heterogeneity, and microenvironmental constraints. Key mechanisms include enhanced DNA repair, disrupted apoptosis, pathway redundancy, altered drug metabolism, oxidative stress tolerance, and glioblastoma stem cell–mediated plasticity. In vivo, resistance is reinforced by the blood–brain barrier, hypoxia, stromal and immune interactions, and selective expansion of therapy-resistant clones. Current strategies to overcome resistance target DNA repair, oxidative stress, autophagy, and metabolic vulnerabilities; however, their efficacy is limited by tumor heterogeneity and delivery barriers. Precision oncology approaches are hampered by a paucity of validated predictive biomarkers, leaving many patients without actionable targets. Ex vivo functional drug sensitivity testing of patient-derived tumor cells offers a complementary strategy, directly assessing individual tumor responses and guiding rational combination therapies. This review highlights the molecular and cellular mechanisms underlying chemoresistance in GBM, examines emerging therapeutic strategies, and explores the potential of integrating personalized, functionally guided approaches into clinical management. Addressing GBM’s profound heterogeneity and adaptive plasticity is essential to improving outcomes in this aggressive and refractory malignancy.

## 1. Introduction

Glioblastoma (GBM), which is classified as a grade 4 tumor, is the most aggressive type of glioma group with poor prognosis for patients. It is characterized by rapid invasive growth, high mitotic activity, microvascular proliferation and necrosis. GBM is the most common primary malignant brain tumor according to the Central Brain Tumor Registry of the United States (CBTRUS) which also demonstrates the lowest 5-year survival rate [1].

**Figure 1:**
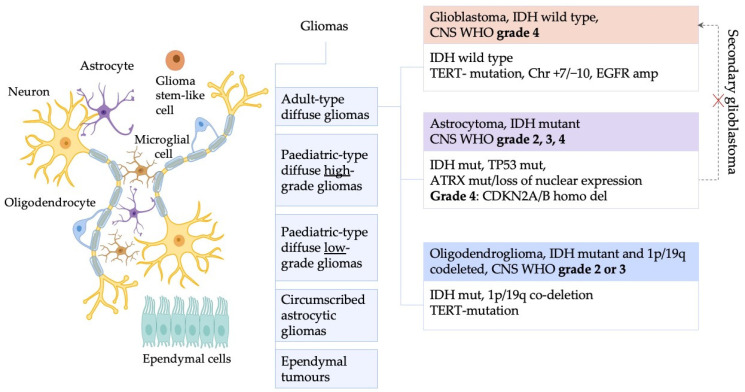
Molecular markers in adult-type diffuse gliomas. Glioblastoma, IDH wild type, grade 4: TERT promoter mutation, EGFR amplification, gain of chromosome 7/loss of chromosome 10. Astrocytoma, IDH mutant, grade 2,3: ATRX and TP53 mutation. CDKN2A/B homozygous deletion—CNS WHO grade 4 astrocytoma. Oligodendroglioma IDH-mutant, 1p/19q co-deleted: TERT promoter mutation (figure created with BioRender.com/5zxjkew, accessed on 26 November 2025).

According to WHO 2021 classification [2], gliomas may be divided into five subgroups: adult-type diffuse gliomas; pediatric-type diffuse low-grade gliomas; pediatric-type diffuse high-grade gliomas; circumscribed astrocytic gliomas and ependymal tumors (Figure 1). The key difference between the former classification and the WHO 2016 classification lies in the absence of the terms “Glioblastoma multiforme, IDH mutant” and “Anaplastic astrocytoma, IDH wild type”, which were known as “secondary glioblastoma”. Adult-type diffuse gliomas account for 90% of all gliomas and are classified into three types: astrocytoma with IDH mutation, oligodendroglioma with IDH mutation and 1p/19q codeletion, and glioblastoma with wild-type IDH. IDH mutations are typically associated with better prognosis [2].

Beyond diagnostic markers, several molecular alterations are commonly associated with gliomas. Homozygous deletion of CDKN2A/CDKN2B characterizes astrocytoma, IDH-mutant, grade 4 and is linked to poor prognosis. ATRX mutations correlate with IDH and TP53 mutations and are mutually exclusive with 1p/19q codeletion, a key biomarker of IDH-mutant astrocytomas (grades 2–4) [3]. GBM’s common alterations are associated with aggressive tumor behavior such as chromosome 7 gain with chromosome 10 loss (+7/−10), TERT promoter mutations, and EGFR amplification [4]. Although MGMT promoter methylation lacks diagnostic value, it serves as an important predictive biomarker for therapy selection [5]. This review focuses on GBM, emphasizing strategies to overcome its pronounced chemoresistance.

GBM remains a major clinical challenge due to its poor prognosis—median patient survival rarely exceeds 14.6 months after diagnosis, even with current standard-of-care interventions that combine surgical resection, radiotherapy, and chemotherapy. GBM resistance to therapy is driven by a broad spectrum of molecular and cellular mechanisms that limit the efficacy of standard treatment modalities. In recent years, extensive data have elucidated the underlying basis of these mechanisms, enabling the development of novel therapeutic approaches.

Several factors limit the efficacy of GBM treatment. Surgical resection is constrained by the tumor’s highly infiltrative growth, preventing complete removal; even after apparently radical surgery, residual cells drive recurrence [4,5]. Reoperation at relapse is often not feasible because of tissue damage from prior surgery or radiotherapy, scarring, edema, or tumor location in eloquent brain regions, where further surgery risks neurological deficits [5].

Radiotherapy remains a cornerstone of GBM therapy, providing improved local control and overall survival but it is not curative. Its effectiveness is limited by infiltrative growth, efficient DNA repair, and metabolic adaptation of tumor cells [6]. Chemotherapy likewise provides no cure but offers a modest yet significant survival benefit [7]. The profound chemoresistance of GBM cells therefore represents a major clinical challenge.

This review examines the main mechanisms driving GBM chemoresistance and discusses current and emerging strategies to overcome it, emphasizing the need for personalized therapeutic approaches.

## 2. Key Chemotherapy Modalities for GBM

### 2.1. Cytotoxic Chemotherapy

Despite extensive research and multiple clinical trials, the standard chemotherapy for GBM has remained largely unchanged. For newly diagnosed GBM, the standard of care after surgery is chemoradiotherapy with temozolomide (TMZ), which increased median overall survival from 12.1 to 14.6 months [8]. TMZ is an alkylating agent that induces DNA methylation, leading to DNA damage, cell cycle arrest, and apoptosis. Its lipophilicity allows effective blood–brain barrier penetration and oral administration [9].

At recurrence, treatment commonly relies on bevacizumab-based regimens, used alone or combined with irinotecan, lomustine, or carboplatin [10,11]. Bevacizumab, an anti-VEGF antibody, can increase median overall survival to approximately 9 months [12], although benefits are limited. While progression-free survival improves (about 4 vs. 1 month), overall survival is not significantly extended [12,13].

Lomustine, an alkylating agent, is widely used as a second-line therapy after progression on TMZ, with a reported median survival of about 9 months [14,15]. Combination regimens containing lomustine, such as PCV, show moderate efficacy in GBM, achieving patient survival of up to 6 months [16].

Carmustine demonstrates greater efficacy when administered locally due to poor blood–brain barrier penetration. Clinically, it is delivered via Gliadel^®^ wafers (Eisai Inc. USA, NJ, Nutley) after surgical resection, improving local tumor control in newly diagnosed patients, though benefits at recurrence are limited and associated with serious adverse events [17,18,19,20].

Irinotecan (CPT-11) is a topoisomerase I inhibitor that is hydrolyzed to its active metabolite, SN-38. SN-38 stabilizes the topoisomerase I–DNA cleavage complex, thereby blocking DNA religation and inducing the replication-associated conversion of single-strand lesions into double-strand breaks that culminate in apoptotic cell death [21]. In recurrent GBM, irinotecan monotherapy provides limited benefit, with a median overall survival of about 14.4 weeks [22]. In contrast, combination therapy with irinotecan and bevacizumab has shown improved outcomes, achieving a median overall survival of approximately 8–9 months [23,24,25].

Carboplatin is a platinum-based agent that forms covalent DNA adducts, mainly intrastrand lesions and interstrand crosslinks, which disrupt DNA structure, inhibit replication and transcription, and induce apoptosis [26]. It is often preferred in neuro-oncology because it more effectively penetrates the disrupted blood–brain barrier within tumors [27].

In recurrent GBM, carboplatin monotherapy shows modest but clinically relevant activity, with responses or disease stabilization in some patients and a median survival of 1.9 months [28,29]. Combination with bevacizumab improves efficacy, extending median survival to 6.9 months [30]. Although, local delivery via convection-enhanced infusion has yielded a median survival of 9.6 months, these findings are based on small cohorts and require confirmation in larger studies [31].

Taken together, these data indicate that the efficacy of currently available chemotherapy regimens remains limited, underscoring the urgent need to develop new therapeutic strategies for GBM.

### 2.2. Targeted Therapy

Targeted cancer therapy is based on the specific inhibition of components of oncogenic driver signaling cascades. In GBM, receptor tyrosine kinases such as EGFR, PDGFR, MET, and VEGFR are frequently activated through gene amplification or gain-of-function mutations, making them attractive targets for molecular therapies.

Approximately 60% of GBMs harbor EGFR amplification or activating mutations [32], prompting numerous attempts to deploy EGFR-targeted agents in GBM treatment [33]. Nevertheless, most EGFR-directed therapies have shown limited clinical benefit [34], largely due to poor blood–brain barrier penetration and rapid tumor cell adaptation. Osimertinib, a third-generation EGFR inhibitor with improved BBB permeability, has demonstrated survival benefits in recurrent GBM with EGFR amplification and EGFRvIII mutations [35,36].

Inhibition of PDGFR with imatinib has not produced meaningful therapeutic benefit in GBM despite promising biological activity in preclinical models [37,38,39]. Phase II clinical trials in recurrent and newly diagnosed GBM showed limited efficacy and no significant survival improvement, likely due in part to poor blood–brain barrier penetration. Other PDGFR inhibitors, including ponatinib [40], dasatinib [41], and tandutinib [42], have likewise failed to demonstrate clinical benefit in GBM. In contrast, the PDGFR inhibitor avapritinib has shown encouraging activity in pediatric GBM with PDGFR alterations [43,44] and in GBM with an MDM2:PDGFRA fusion [45] with objective responses observed in a subset of patients. The success of avapritinib may be attributed to its ability to cross the blood–brain barrier and the use of molecularly stratified treatment selection.

The receptor tyrosine kinase MET and its ligand HGF are frequently hyperactivated in gliomas through overexpression, gene amplification, oncogenic fusions (e.g., PTPRZ1–MET), or autocrine HGF signaling [46]. Several MET inhibitors, including capmatinib, tepotinib, and cabozantinib, have shown limited efficacy in unselected populations but confer clinical benefit in molecularly stratified patients with confirmed MET alterations [47,48,49]. Notably, the PTPRZ1–MET fusion is associated with aggressive disease and represents a promising predictive biomarker [50,51].

Dysregulation of the RTK/RAS/RAF/MEK/ERK pathway is a key driver of GBM growth, survival, invasion, and radioresistance. Although RAS mutations are rare, pathway activation commonly results from EGFR amplification, NF1 loss, or RTK–PI3K/AKT crosstalk [52]. Accordingly, pharmacological targeting of RAS signaling is biologically justified; however, farnesyltransferase inhibitors such as tipifarnib and lonafarnib have demonstrated minimal clinical activity as monotherapies in recurrent GBM, with responses limited to small patient subsets [53,54,55]. Activating BRAF mutations, predominantly BRAF V600E, occur in approximately 3% of GBM cases and provide a rationale for targeted therapy. Selective BRAF inhibitors, alone or in combination with MEK inhibitors, have shown clinical efficacy in BRAF-mutant GBM, with reports of durable responses, particularly in epithelioid variants [56,57,58,59].

Inhibition of the PI3K/mTOR pathway using everolimus or temsirolimus has yielded modest overall efficacy. However, molecular stratification identifies responsive subgroups, including tumors with mTORSer2448 phosphorylation or TSC2 mutations [60,61,62]. Temsirolimus activity correlates with mTOR pathway activation, while everolimus has demonstrated benefit in individual TSC2-mutant cases. Thus, biomarkers of PI3K/mTOR signaling activation (e.g., phospho-mTOR, phospho-S6, phospho-p70S6K, 4EBP1) and PTEN/PI3K/AKT/EGFR status may guide patient selection for targeted therapy.

Alterations activating the CDK4/6–Rb–E2F axis, including CDKN2A deletion, CDK4 amplification, and RB1 dysregulation, are common in GBM and promote sustained proliferation [63], providing a rationale for CDK4/6 pathway inhibition. However, clinical trials of CDK4/6 inhibitors (ribociclib, palbociclib, abemaciclib) have produced mixed results. Although these agents penetrate tumor tissue and effectively inhibit Rb phosphorylation [64,65], ribociclib and palbociclib have shown minimal clinical benefit as monotherapies in recurrent disease [64,65,66]. In contrast, abemaciclib has prolonged progression-free survival in molecularly selected patients with intact Rb signaling and activating pathway alterations [67]. These data highlight the importance of molecular stratification based on RB1 status and upstream regulators (CDKN2A, CDK4). Tumors with RB1 loss are intrinsically resistant to CDK4/6 inhibition and should not be treated with these agents [68].

Combination strategies that simultaneously inhibit multiple oncogenic cascades, such as PDGFR inhibitors combined with EGFR, MET or mTOR inhibitors, or CDK4/6 inhibitors combined with mTOR inhibition or standard chemo-radiotherapy, have demonstrated synergistic activity in preclinical models [69,70].

Despite compelling preclinical evidence for numerous targeted agents, the translation of these therapies into meaningful clinical benefit in GBM patients remains limited. One of the foremost barriers is the blood–brain barrier, which restricts the entry and adequate distribution of most small molecules and large biologics into the central nervous system, often preventing therapeutically effective concentrations at the tumor site despite promising in vitro activity [71]. Moreover, GBMs exhibit profound intratumoral heterogeneity, both phenotypically and genetically, such that distinct subclones within the same tumor can respond differently to a given molecular inhibitor, enabling resistant populations to drive therapeutic failure and recurrence.

Finally, many clinical trials to date have lacked molecularly stratified patient selection, diluting potential benefits in responsive subgroups and obscuring true efficacy; without predictive biomarkers to guide enrollment, agents with activity in defined genetic contexts often show modest results in unselected cohorts.

These findings underscore the critical importance of biomarker-driven patient stratification in trials of targeted therapies. Lack of molecular selection can mask efficacy within responsive subgroups, even when no benefit is evident in unselected populations. Personalized therapy can mitigate key limitations of targeted treatment in GBM by enabling molecular stratification of patients and selection of agents directed against specific oncogenic drivers with adequate blood–brain barrier penetration. This approach helps address intratumoral heterogeneity and signaling pathway redundancy by guiding rational mono- or combination therapies, thereby increasing the likelihood of clinical benefit.

### 2.3. Immunotherapy

Immunotherapy, e.g., encompassing immune checkpoint blockade, dendritic cell vaccines, strategies targeting immunosuppressive components of the tumor microenvironment (TME), has become an important modality of personalized cancer therapy. However, its application in GBM remains challenging due to the profoundly immunosuppressive, “cold” nature of the GBM TME, necessitating careful patient selection. The immunologically ‘cold’ nature of the GBM TME stems from multiple mechanisms, including physical barriers such as the blood–brain barrier, low neoantigen burden, and metabolic constraints such as glucose deprivation and lactate accumulation. Immunosuppressive cellular components including M2-polarized TAMs, regulatory T-cells, and MDSCsб secrete inhibitory cytokines (e.g., TGF-β, IL-10) and express checkpoint molecules (PD-L1, CTLA-4), collectively limiting T-cell infiltration, activation, and effector function.” While this Review focuses on mechanisms of chemoresistance and the role of tumor stem cells, emerging insights into GBM immunity are critical; we refer the reader to dedicated reviews on the GBM TME and immunotherapeutic limitations [72,73].

Despite testing various immunotherapeutic strategies, durable responses in GBM remain elusive, with pivotal trials of immune checkpoint blockade, CAR-T cells, and vaccine platforms showing limited survival benefit. GBM drives profound systemic and local immunosuppression, marked by lymphopenia, T-cell sequestration in bone marrow [74], and TME dominance by immunosuppressive tumor-associated macrophages (TAMs), regulatory T-cells, and myeloid-derived suppressor cells (MDSCs) [75,76]. Infiltrating T-cells undergo apoptosis, anergy, exhaustion, and senescence due to suppressive signals and metabolic constraints [77].

The efficacy of immunotherapy in GBM is also limited by the blood–brain barrier, profound tumor heterogeneity, and therapy-induced phenotypic plasticity—particularly a shift toward more aggressive mesenchymal states that stimulate a hypoxic, immunosuppressive TME. Recent single-cell and multiomic studies have begun to decode this complexity, revealing dynamic immune remodeling and resistance pathways not captured by conventional profiling [78].

## 3. Major Mechanisms of GBM Resistance to Chemotherapy

Glioblastoma exhibits profound resistance to chemotherapy, reflecting a convergence of intrinsic tumor cell properties and adaptive, therapy-induced mechanisms that emerge in vivo.

At the cellular level, GBM resistance is driven by upregulation of DNA damage repair pathways, disruption of apoptotic signaling, signaling redundancy and plasticity, altered drug transport and metabolism, and enhanced tolerance to oxidative stress. Many of these mechanisms are cell-autonomous and can be observed in in vitro GBM models, including established cell lines and patient-derived cultures, where they represent fundamental survival strategies under uniform cytotoxic or targeted therapeutic pressure [79,80,81].

However, therapeutic resistance in patients extends beyond intrinsic cellular adaptations and is critically shaped by the tumor microenvironment and anatomical constraints unique to the central nervous system. In vivo, blood–brain barrier–mediated drug exclusion, intratumoral heterogeneity, hypoxia, metabolic gradients, stromal interactions, immune suppression, and clonal selection under treatment collectively drive adaptive and acquired resistance. While intrinsic mechanisms such as MGMT-mediated DNA repair can confer primary resistance to alkylating agents, therapy-induced alterations in DNA repair capacity, cellular state, and immune evasion contribute to disease progression and recurrence.

### 3.1. Upregulation of DNA Repair Processes

Since the primary mechanism of most cytotoxic chemotherapeutic agents used in GBM treatment involves the induction of DNA damage in tumor cells, alterations in DNA repair systems represent a principal factor underlying GBM chemoresistance.

Alkylating agents such as temozolomide, lomustine, and carmustine induce DNA lesions including N7-methylguanine, N3-methyladenine, and O6-methylguanine, with N7-methylguanine and N3-methyladenine primarily repaired by the base excision repair (BER) pathway. BER involves lesion recognition by DNA glycosylases, formation of apurinic/apyrimidinic sites, strand cleavage by AP endonuclease, gap filling by DNA polymerase, and ligation by DNA ligase [82]. PARP1 plays a central role in BER by sensing single-strand breaks and promoting recruitment of repair factors through poly(ADP-ribosyl) [83]. Upregulation of BER components, including DNA glycosylases and PARP1, has been associated with the development of TMZ resistance and poor prognosis in GBM [84,85,86].

O6-methylguanine (O6-meG) is a small DNA adduct that does not markedly distort the DNA helix and is therefore poorly recognized by the base excision repair (BER) machinery, which primarily detects bulkier lesions such as N7-meG and N3-meA. As a result, BER does not contribute to O6-meG repair [38]; instead, this lesion is removed by O6-methylguanine-DNA methyltransferase (MGMT), a direct reversal enzyme that eliminates O6-alkyl groups from guanine [39]. High MGMT expression is a major driver of resistance to TMZ and other alkylating agents in GBM. MGMT transcription is suppressed by promoter methylation, which is strongly associated with improved response to TMZ and prolonged survival [40,41]. Accordingly, MGMT promoter methylation status is an established prognostic biomarker in GBM, with median overall survival of 21.2 months in methylated versus 14 months in unmethylated tumors [42].

Mismatch repair (MMR) is a key pathway involved in the response to DNA damage induced by alkylating agents. It functions through recognition of mismatched bases by MutS proteins, recruitment of MutL complexes, excision of the error-containing DNA strand, and subsequent DNA resynthesis and ligation [87]. The role of MMR in the TMZ resistance in GBM is dual. MMR deficiency allows tumor cells to tolerate TMZ-induced mismatches, promoting mutation accumulation, cell survival, and resistance [88,89]. In contrast, an intact MMR system can enhance TMZ cytotoxicity through futile repair cycles at O6-MeG:T mismatches in which O6-MeG is mispaired with thymine and MMR preferentially removes the thymine base rather than O6-MeG, ultimately leading to DNA double-strand breaks and apoptosis [88,89,90].

Topoisomerase I–DNA crosslinks formed in response to irinotecan are repaired by tyrosyl-DNA phosphodiesterase 1 (TDP1), which releases the DNA 3′ end and enables subsequent repair by the base excision repair (BER) pathway [91]. The resulting DNA double-strand breaks are resolved by homologous recombination (HR) and non-homologous end joining (NHEJ). Accordingly, increased activity of TDP1 and the BER, HR, and NHEJ pathways contributes to resistance to irinotecan [92,93].

Intrastrand platinum adducts formed in response to carboplatin are removed by nucleotide excision repair, primarily through the ERCC1–XPF endonuclease complex, whose increased expression is associated with resistance to platinum-based therapies [94,95]. HR is required to repair replication-associated double-strand breaks caused by platinum lesions, and HR deficiencies such as BRCA mutations sensitize tumors to carboplatin [96].

### 3.2. Disruption of Apoptotic Signaling

Disruption of apoptotic signaling is another critical mechanism by which GBM cells evade chemotherapy, driven by imbalanced Bcl-2 family proteins, p53 pathway inactivation, and hyperactivation of pro-survival pathways such as PI3K/AKT/mTOR. Anti-apoptotic proteins Bcl-2, Bcl-xL, and Mcl-1 are upregulated in GBM, shifting the balance away from cell death [97,98]. The p53 pathway is altered in approximately 85% of GBMs through mutations in TP53 or dysregulation of CDKN2A/ARF and MDM2/MDM4, leading to impaired p53 function [99]. Loss of ARF or overactivation of MDM2 promotes p53 degradation [100], while PI3K/AKT signaling further suppresses p53 and the pro-apoptotic protein Bad [101]. In addition, mutant p53 can acquire oncogenic functions that activate pro-inflammatory signaling and drive tumor progression [102].

### 3.3. Pathway Redundancy

GBM is a tumor with biological features that distinguish it from many systemic cancers that are driven predominantly by single receptor tyrosine kinase (RTK) oncogenes (e.g., EGFR mutations in non-small-cell lung cancer). GBM exhibits extensive molecular heterogeneity, plasticity and pathway complexity, with frequent co-activation of multiple RTKs, including EGFR, PDGFR, MET and others, within the same tumor. This co-activation results in redundancy and compensation among signaling pathways, such that inhibition of any single RTK fails to shut down oncogenic signaling effectively.

The simultaneous activation of multiple signaling pathways in GBM is not incidental but reflects a convergence of developmental lineage context, genomic architecture, and network-level deregulation that is uncommon in most other solid tumors. This phenomenon arises in part from the absence of a single dominant oncogenic driver in GBM; instead of oncogene addiction, GBM evolves under selective pressure to maintain signaling robustness through redundancy. Epigenetically permissive chromatin landscape of GBM cells, which resembles that of neural progenitor cells and enables broad expression of multiple growth factor receptors and signaling intermediates. This permissiveness allows GBM cells to transcriptionally sustain multiple signaling programs at once, rather than committing to a single lineage-restricted signaling axis [103].

### 3.4. Modulation of Transmembrane Transport Processes

Transmembrane transport processes critically influence the intracellular accumulation and cytotoxicity of chemotherapeutic agents, and their dysregulation represents an important mechanism of chemoresistance in GBM. ATP-binding cassette (ABC) transporters actively export anticancer drugs from tumor cells, reducing intracellular drug levels and therapeutic efficacy [104]. The most extensively studied ABC transporters in GBM include ABCB1 (P-glycoprotein), ABCC1, and ABCG2 [104,105]. Elevated activity of these transporters confers resistance to multiple agents, including TMZ [106,107], carboplatin, carmustine [108], and irinotecan [109].

### 3.5. Oxidative Stress Resistance

Another mechanism contributing to GBM chemoresistance is the capacity of tumor cells to tolerate oxidative stress. TMZ [110], platinum-based agents, irinotecan [109], and many other chemotherapeutics promote the generation of reactive oxygen species (ROS) within tumor cells. Accumulation of ROS triggers oxidative stress and cell death, mediating the cytotoxic effects of these drugs. In GBM, robust antioxidant defense systems counterbalance this oxidative burden, and heightened activity of these systems is directly associated with chemoresistance [111,112,113]. Glioma cells resistant to TMZ display lower intracellular ROS levels, increased glutathione levels, upregulated glutathione reductase [114], and higher expression of the glutamate–cystine transporter xCT [114,115]. Moreover, higher expression of the xCT component, thioredoxin reductase and thioredoxin has been significantly correlated with clinical outcomes, including shorter overall and progression-free survival [115,116,117].

### 3.6. Drug Metabolism

Chemotherapy resistance can also arise from tumor-specific drug metabolism. Irinotecan, a prodrug, requires conversion to its active metabolite SN-38 by carboxylesterases. Reduced expression or activity of these enzymes in GBM can limit SN-38 formation and promote resistance [118]. In addition, enhanced inactivation of SN-38 via glucuronidation by UGT1A isoforms (including UGT1A1 and UGT1A10) further lowers active drug levels. Upregulation of UGT1A enzymes, either within the tumor or systemically, thereby contributes to irinotecan resistance [119].

### 3.7. Blood–Brain Barrier

A defining challenge in vivo is the restrictive nature of the blood–brain barrier, which limits penetration of many systemic therapies into tumor tissue, even in regions of contrast enhancement where the barrier is partially compromised [120]. Efflux transporters, such as ABCB1, ABCG2 and other ABCC family members that transport substrates out of the endothelium and back into the circulation, further reduce effective intratumoral drug concentrations, fundamentally altering drug exposure compared to in vitro systems [121].

### 3.8. The Tumor Microenvironment

TME plays a central role in GBM chemoresistance by establishing hypoxia, acidosis, and extensive interactions with non-neoplastic cells, including vascular, neural, microglial, and immune compartments, all of which critically shape therapy response [122]. These conditions regulate angiogenesis, immune evasion, apoptosis, DNA repair, oxidative stress, and the expression and activity of ABC transporters [123]. Hypoxia, in particular, through HIFα-mediated signaling, suppresses pro-apoptotic pathways and promotes a stem-like tumor phenotype associated with resistance [124]. Reciprocal interactions between GBM cells and TME-resident non-tumor cells further drive tumor progression toward more aggressive and therapy-resistant states [125,126,127,128]. Tumor-associated macrophages and microglia, which can constitute up to 50% of the immune infiltrate, adopt immunosuppressive phenotypes and secrete cytokines that support GBM growth and invasion [129]. The adaptive immune compartment contains limited populations of CD8^+^ cytotoxic and CD4^+^ helper T-cells, as well as NK cells, which are frequently rendered dysfunctional or exhausted within the immunosuppressive milieu [130], while myeloid-derived suppressor cells and dendritic cells further reinforce local and systemic immune suppression [122]. In parallel, endothelial cells and pericytes promote aberrant angiogenesis and form perivascular niches that sustain glioma stem cells (GSCs) and reinforce resistance [131], while astrocytes and neuronal elements engage in bidirectional signaling with GBM cells, modulating invasion, metabolism, and immunoregulation [122]. Ultimately, a population of glioblastoma stem cells residing in hypoxic and perivascular niches act as key organizers of the TME, maintaining cellular plasticity and driving intratumoral heterogeneity and therapeutic resistance [132].

### 3.9. Immune Suppression

GBM develops within a strongly immunosuppressive TME that impairs effective antitumor immune responses. GBM cells secrete immunosuppressive mediators such as TGF-β and IL-10, promote regulatory T cell expansion, and drive tumor-associated macrophages toward an M2-like phenotype, collectively inhibiting cytotoxic T cell activity [133]. In addition, poor antigen presentation and high expression of immune checkpoint molecules, including PD-L1, contribute to T cell exhaustion and resistance to immunotherapy [134]. Systemic immune dysfunction and cytokines such as IL-6 further reinforce immune suppression, highlighting key barriers to effective immunotherapeutic strategies in GBM [135].

The immunosuppressive tumor microenvironment in GBM has been implicated in reduced efficacy not only of immunotherapies but also of standard treatments such as radiotherapy and TMZ chemotherapy. Conventional therapies can induce systemic immunosuppression and modulate local immune phenotypes, while the baseline inhibitory milieu supports tumor survival and therapy resistance [136,137]. Baseline immune characteristics in GBM, including patterns of immune cell infiltration and systemic immune markers such as neutrophil-to-lymphocyte ratio, have significant prognostic implications, with certain immunological signatures and higher levels of specific immune populations correlating with overall survival. These immune profiles can serve as independent prognostic tools in risk stratification [138,139].

### 3.10. Conclusions

Despite extensive characterization of chemoresistance mechanisms in GBM, only a limited subset is currently amenable to clinical targeting. MGMT promoter methylation remains the only validated predictive biomarker incorporated into clinical guidelines, guiding the use of alkylating agents such as TMZ [140]. In addition, recent therapeutic guidelines for CNS tumors recognize BRAF p.V600E as an effectively actionable target and recommend integrating molecular screening into standard care to identify targetable genetic variants prior to therapy selection, particularly in rare tumor entities or when no further standard treatment options are available [2]. Nevertheless, other mechanisms, clearly contributing to resistance, are not yet therapeutically exploited in GBM due to toxicity, pathway redundancy, and limited therapeutic windows.

For cytotoxic chemotherapy, resistance is primarily driven by MGMT expression, efficient or adaptive DNA repair, apoptotic pathway dysregulation, and restricted drug delivery across the blood–brain barrier. These factors underlie the modest survival benefit of TMZ and inevitable recurrence. In targeted therapies, resistance is dominated by pathway redundancy, signaling plasticity, and epigenetic permissiveness. The lack of a single dominant oncogenic driver enables GBM cells to sustain parallel signaling programs, limiting the efficacy of targeted agents and their routine clinical use. For immunotherapies, resistance is largely dictated by the immunosuppressive tumor microenvironment, characterized by impaired antigen presentation, immune checkpoint expression, macrophage polarization, regulatory T-cell expansion, and systemic immune dysfunction. Across all therapeutic modalities, cytotoxic, targeted, and immune-based, blood–brain barrier and profound intratumoral heterogeneity represent universal limitations, restricting durable responses.

In summary, GBM resistance reflects layered intrinsic and acquired mechanisms, only a fraction of which are currently actionable. This highlights the need for personalized GBM treatment strategies integrating molecular and functional profiling to guide rational therapeutic selection.

## 4. GBM Heterogeneity as a Driver of Chemoresistance

Inter- and intratumoral heterogeneity is a key contributor to the pronounced GBM resistance to therapy. Advancements in high-throughput omics technologies have enabled comprehensive profiling of tumor cell populations in patient samples, revealing key genetic, epigenetic, transcriptomic, and metabolic GBM subtypes [141].

### 4.1. Complex Genetic Landscape of GBM

At the genetic level, GBM heterogeneity primarily arises from driver mutations affecting signaling pathways that control proliferation, cell cycle progression, stress responses, and apoptosis. Genetic alterations present in 80–90% of GBMs converge mainly on three core pathways. Disruption of the p53-mediated apoptotic axis through mutations in TP53, MDM2, and MDM4 impairs cell death signaling. Concurrently, alterations in cell cycle regulators (CDKN2A/B, CDK4, CDK6, CCND2, RB1), amplification or activating mutations of growth factor receptors (e.g., EGFR, PDGFR), and loss of the PTEN tumor suppressor drive uncontrolled proliferation and activate MAPK and PI3K/AKT signaling, thereby promoting tumor survival under stress conditions [32].

A major source of genetic heterogeneity in GBM is chromosomal aneuploidy, particularly the combined gain of chromosome 7 and loss of chromosome 10 (+7/−10 genotype), observed in approximately 79% of cases [90]. Chromosome 10 loss often represents an early oncogenic event and leads to deletion of PTEN, a key negative regulator of RAS/MAPK/ERK signaling [91], along with additional tumor suppressors such as ANXA7, ADARB2, and KLF6 [92,93]. In contrast, chromosome 7 polysomy may act as a compensatory mechanism supporting tumor cell viability through increased dosage of oncogenes, including EGFR, MET, BRAF, and PDGFA [94,95]. These copy number alterations constitute a dominant driver of inter- and intratumoral molecular heterogeneity and are incorporated into the current WHO classification of CNS tumors as diagnostic markers [2]. The coexistence of multiple cooperating driver mutations generates tumor-specific genomic profiles, underscoring the need for personalized therapeutic strategies.

EGFR frequently undergoes structural alterations in GBM. As a receptor tyrosine kinase, EGFR activates key oncogenic signaling cascades, including RAS/MAPK/ERK, PI3K/AKT, JAK/STAT, and PLCγ/PKC, which regulate cellular proliferation, metabolism, and survival. In GBM, aberrant EGFR signaling maintains stem-like properties, enhances stress tolerance, promotes therapeutic resistance, and drives invasion, angiogenesis, and inflammation [142]. EGFR amplification and structural rearrangements, such as activating extracellular domain mutations and exon deletions that stabilize the active receptor conformation, occur in more than 57% of primary GBMs [32]. The most prevalent alteration is deletion of exons 2–7, generating the ligand-independent, constitutively active EGFRvIII variant [143]. In addition to canonical EGFR pathways, EGFRvIII activates a broader network of receptor tyrosine kinases and downstream effectors, further enhancing tumor growth, extracellular matrix remodeling, angiogenesis, and resistance to therapy [143]. Co-occurrence of multiple EGFR alterations within single tumors contributes to pronounced intra- and intertumoral heterogeneity [144].

### 4.2. Epigenetic Heterogeneity of GBM

Epigenetic mechanisms, including DNA methylation, histone modifications, chromatin remodeling, and noncoding RNA regulation, substantially contribute to GBM heterogeneity. Profiling of chromatin-associated proteins indicates that aberrant enhancer epigenetic states are associated with more aggressive GBM phenotypes [145]. In addition, the H3.3 K27 point mutation is linked to particularly poor prognosis [146]. Epigenetic dysregulation alters proliferation and tumor immunogenicity by modulating oncogenic drivers [147], leading to widespread changes in gene expression, signaling pathways, and control of the cell cycle, apoptosis, and DNA repair [148]. Clinically, GBM recurrence is often accompanied by loss of MGMT promoter methylation, which confers increased resistance to TMZ [149]. TMZ resistance is further reinforced by siRNA-mediated upregulation of ABC transporters and induction of stem-like cellular states [123,150]. Together, genetic and epigenetic alterations drive extensive transcriptomic, proteomic, metabolic, and phenotypic heterogeneity in GBM [147].

### 4.3. GBM Transcriptomic Heterogeneity

GBM exhibits pronounced transcriptomic heterogeneity closely linked to its mutational landscape. Gene expression profiling has defined three major transcriptomic subtypes: proneural, classical, and mesenchymal, each associated with distinct driver alterations [125,151]. The proneural subtype is characterized by PDGFRA, IDH1, and TP53 mutations and expression of neural progenitor markers, whereas the classical subtype is defined by EGFR amplification and astrocytic markers. The mesenchymal subtype is associated with NF1 or TP53 mutations and mesenchymal lineage markers. The latter is linked to the most aggressive clinical behavior, and poor prognosis [152,153,154]. Although proneural GBMs generally show more favorable outcomes, recurrent tumors frequently acquire resistance and undergo a transcriptomic shift toward a mesenchymal-like phenotype with stronger angiogenic, and hypoxic potential [125,126,155]. Indeed, 45–49% of recurrent GBMs initially classified as proneural or classical transition to the mesenchymal subtype, limiting the efficacy of first-line therapies [125,156]. Proteomic profiling further revealed GBM subsets driven by KRAS, MYC, or HIF1α signaling, with KRAS-associated tumors displaying mesenchymal traits and poor prognosis, whereas MYC-driven tumors preferentially exhibited proneural characteristics [157].

### 4.4. Metabolic Heterogeneity

Additional classifications based on metabolic and signaling pathway activity identified glycolytic/plurimetabolic, mitochondrial, neuronal, and proliferative/progenitor subtypes, which largely overlap with established transcriptomic and cellular states [158]. Notably, patients with the mitochondrial subtype exhibit significantly improved survival. Complementary metabolic flux analyses distinguished glycolytic, oxidative, and mixed metabolic subtypes, linking glycolysis to mesenchymal features and oxidative metabolism to neurogenic programs and radiosensitivity [158,159].

### 4.5. Intratumoral Heterogeneity in GBM

Advances in single-cell genomics have delineated the complex landscape of glioblastoma, demonstrating that multiple transcriptional and phenotypic cell populations coexist within the same tumor, driving adaptation and chemoresistance. Multi-regional single cell analysis has demonstrated that tumor cells with distinct transcriptional profiles may coexist within the same lesion [160], with individual cells exhibiting phenotypic characteristics of more than one molecular subtype [161], reflecting a high level of intra-tumoral heterogeneity.

Single-cell transcriptomic analyses further refined GBM heterogeneity by identifying four cellular states: astrocyte-like (AC-like), oligodendrocyte precursor-like (OPC-like), neural progenitor-like (NPC-like), and mesenchymal-like (MES-like) [127]. AC-like cells largely correspond to the classical subtype, OPC-like and NPC-like cells align with the proneural subtype, and MES-like cells closely match the mesenchymal transcriptomic profile, expressing genes involved in mesenchymal transition, hypoxia adaptation, glycolytic metabolism, extracellular matrix remodeling, and inflammation [125,151,162].

Intratumoral heterogeneity not only defines distinct molecular and phenotypic states within GBM but also contributes to chemoresistance by allowing sensitive and resistant clones to coexist within the same tumor [163]. Single-cell, spatial multi-omics and longitudinal analyses have shown that GBM harbors subpopulations with differing susceptibility to therapy, such that pre-existing resistant clones survive cytotoxic stress and expand under treatment, while sensitive cells are eliminated [164,165,166]. This selection of resistant subclones, together with phenotypic plasticity that enables transitions between cell states, contributes to treatment failure and recurrence in patients with GBM [167].

In addition, pronounced intratumoral heterogeneity, with coexisting EGFR variants and resistant subclones, further compromises the efficacy of EGFR-targeted therapies [144]. Resistance commonly arises through loss of EGFRvIII encoded on extrachromosomal DNA during treatment, followed by restoration after therapy termination [168,169].

### 4.6. Cellular Plasticity of GBM

GBM exhibits pronounced cellular plasticity, enabling genetic, transcriptomic, metabolic, and functional reprogramming that fundamentally distinguishes it from many other solid tumors and underlies its aggressive clinical behavior. Rather than following fixed genetic hierarchies, GBM cells undergo dynamic and reversible transitions between stem-like, progenitor-like, and differentiated states, as demonstrated by single-cell transcriptomic analyses [127]. Microenvironmental cues, including hypoxia, inflammatory signaling, and vascular niche interactions, actively promote these transitions, particularly toward mesenchymal and stem-like programs associated with invasion and therapy resistance [125,126,156]. GBM plasticity is enabled in part by an epigenetically permissive chromatin landscape that resembles that of neural progenitor cells, allowing broad transcriptional accessibility and rapid state switching without the need for additional genetic alterations [103,170]. This permissive chromatin architecture facilitates transcriptional reprogramming in response to environmental stress, including therapy, hypoxia, and immune pressure. As a result, therapeutic pressure does not simply select for pre-existing resistant clones, but actively induces adaptive cell-state reprogramming, facilitating rapid tumor evolution and recurrence. These observations highlight the need for therapeutic strategies that target dynamic regulatory networks and microenvironmental interactions in addition to genetic drivers.

Overall, limited efficacy of targeted monotherapies largely reflects pathway redundancy and signaling plasticity. For example, mechanisms of EGFR inhibitor resistance include activation of alternative receptor tyrosine kinases such as MET and PDGFR [36,171] and PTEN loss leading to constitutive PI3K signaling [33,172]. Adaptive resistance to RB1 targeting agents frequently emerges through activation of compensatory pathways, including PI3K/mTOR, c-MET, and Trk signaling [64]. This heterogeneity is compounded by signaling pathway redundancy and plasticity, where inhibition of one oncogenic axis (e.g., EGFR) can be bypassed by alternative receptor tyrosine kinases or downstream effectors, undermining sustained clinical responses [173].

Taken together, GBM displays profound molecular and phenotypic heterogeneity that functions as an active resistance mechanism rather than a descriptive feature, substantially limiting the effectiveness of uniform treatment strategies. Understanding the rules governing tumor cell plasticity and heterogeneity may therefore enable refined molecular stratification and support the development of personalized therapeutic approaches for GBM.

## 5. Glioblastoma Stem Cells in GBM Chemoresistance

Intratumoral heterogeneity in GBM is largely driven by a subpopulation of tumor-initiating stem-like cells. Such cancer stem cells have been identified in multiple malignancies, highlighting their central role in tumor biology [174]. In GBM, glioblastoma stem cells (GSCs) were first described by Singh et al. as a cell population capable of initiating tumors in vivo [175]. GSCs exhibit high tumorigenic potential, self-renewal capacity, and phenotypic plasticity, express neural stem cell markers, and generate differentiated progeny that constitute the bulk of the tumor mass [176].

In addition to genetic selection, GSCs contribute substantially to therapy resistance. GSCs display high plasticity and are capable of surviving standard-of-care treatments such as TMZ and radiotherapy, repopulating tumors after treatment and reconstituting intratumoral heterogeneity. These characteristics suggest that therapy resistance in GBM often reflects a combination of pre-existing resistant cells and dynamic adaptation, mediated by cancer stem-like phenotypes and microenvironmental interactions [177].

The cellular origin of GSC remains incompletely understood; proposed progenitors include neural stem cells, mature astrocytes, oligodendrocyte precursor cells, and perivascular mesenchymal stromal cells [178,179,180]. GSCs persist after standard therapy and drive tumor recurrence due to their intrinsic resistance to treatment [181]. GSCs show pronounced activation of chemoresistance pathways commonly observed in GBM: elevated MGMT expression and DNA repair activity [182], increased ABC transporter function [108], reduced reactive oxygen species levels [183], and dysregulation of apoptotic pathways [132,184].

Within the tumor, GSCs reside in specialized niches that promote their survival, self-renewal, and therapeutic resistance. The principal niches include the perivascular niche, associated with tumor vasculature, and the hypoxic niche surrounding necrotic regions. Hypoxia enhances stemness by activating self-renewal and dedifferentiation programs, thereby conferring resistance to chemo- and radiotherapy [185]. In the perivascular niche, endothelial cells support GSC maintenance and proliferation, while GSCs can transdifferentiate into endothelial cells and pericytes, directly contributing to tumor angiogenesis [186,187].

GSCs display pronounced molecular and genetic plasticity, enabling adaptation to microenvironmental and therapeutic pressures. Anticancer treatment can select for genetically altered, drug-resistant GSC clones that drive tumor recurrence [188]. GSCs are proposed to function as units of clonal evolution, accumulating driver mutations and generating intratumoral heterogeneity at the stem cell level. Notably, proneural GSCs, which are initially therapy-sensitive, can transition to a mesenchymal, resistant phenotype upon recurrence [189].

Importantly, differentiated GBM cells can also undergo epigenetic reprogramming and revert to a stem-like state [190]. Although GSCs can differentiate and exit the cell cycle, this process is reversible, allowing reacquisition of stemness and proliferative capacity [191]. TMZ treatment may further promote dedifferentiation of non-stem GBM cells into GSCs, increasing tumor plasticity and resistance [192,193]. The acquisition of stem-like properties by non-stem tumor cells is not unique to GBM; this phenomenon has been observed across multiple solid malignancies and represents an additional manifestation of cancer cell plasticity [194]. In addition, though stem-like cells possess self-renewal capacity and therapy resistance, maintaining this state is metabolically costly and typically associated with slower proliferation. Differentiation enables functional specialization, rapid expansion of tumor mass, and increased phenotypic heterogeneity, which enhances adaptability to environmental stress and treatment. Thus, rather than maximizing stemness across all cells, tumors benefit from a dynamic balance between self-renewal and differentiation that optimizes both persistence and growth.

The high molecular and genetic plasticity of GSC enables dynamic adaptation to chemotherapeutic stress, positioning them as central determinants of GBM recurrence and treatment resistance [195].

## 6. Co-Occurring and Reinforcing Mechanisms in the Clinical Setting

In patients with GBM, resistance arises from interconnected, adaptive networks rather than isolated mechanisms, with tumor hypoxia, microenvironmental interactions, and cellular plasticity reinforcing each other to sustain therapeutic failure. For example, chronic hypoxia within tumor niches stabilizes HIF-1α signaling, which promotes stem-like phenotypes, angiogenesis, metabolic reprogramming, and DNA damage tolerance, thereby enhancing resistance to radiotherapy, chemotherapy, and antiangiogenic therapy [196]. At the same time, extensive intratumoral heterogeneity including GSCs and mesenchymal transition cooperates with immune suppression driven by tumor-associated macrophages and regulatory T-cells to create an immunosuppressive microenvironment and evade immunotherapies [197]. Limited drug penetration across a variably disrupted blood–brain barrier further exacerbates these effects by reducing effective drug exposure and selecting for resistant clones, while efflux transporter upregulation and epigenetic plasticity (e.g., ECM-associated gene regulation and stemness circuitry) enable persistent survival. Together, these co-occurring processes form a highly redundant and adaptive resistance network that undermines conventional and targeted treatments in GBM patients.

Thus, multiple and diverse mechanisms underlie GBM cell chemoresistance, and the specific drivers of resistance can vary substantially among individual patients. This marked heterogeneity underscores the imperative for personalized therapeutic strategies tailored to the distinct resistance profiles present in each patient.

## 7. Approaches to Overcome Resistance in GBM

To overcome GBM resistance to chemotherapeutic agents, numerous strategies targeting established determinants of drug resistance have been explored.

### 7.1. Targeting DNA Repair

One line of research has focused on sensitizing GBM to therapy by modulating DNA repair pathways. A key determinant of GBM resistance to alkylating agents is the expression of MGMT, and numerous attempts have been made to target this enzyme to reverse resistance. Direct MGMT inhibitors such as O6-benzylguanine and lomeguatrib restore sensitivity to TMZ in preclinical models [198,199], but failed clinically due to limited efficacy, rapid MGMT recovery, and significant hematologic toxicity [200,201,202]. Epigenetic strategies to suppress MGMT expression using HDAC or DNMT inhibitors are conceptually promising, though clinical translation is hindered by limited selectivity and unpredictable effects. Nevertheless, preclinical data suggest that epigenetic modulation can re-sensitize GBM cells to TMZ regardless of baseline MGMT status [203]. SiRNA-mediated MGMT knockdown enhances TMZ sensitivity in preclinical models, but remains clinically untested due to delivery challenges and instability in vivo [204].

Targeting MGMT and DNA repair pathways to overcome resistance is limited by the redundancy and adaptability of DNA damage response networks. Although MGMT is a key mediator of TMZ resistance, its inhibition alone is frequently insufficient due to compensatory activation of MMR, BER, and HR pathways. In addition, MGMT expression is controlled by multiple transcriptional and epigenetic mechanisms beyond promoter methylation, diminishing its predictive value and therapeutic tractability. Intratumoral heterogeneity further promotes adaptive resistance through the coexistence of MGMT-high and MGMT-low subclones [80,81].

PARP inhibitors such as olaparib, veliparib, and niraparib represent a promising strategy to overcome GBM chemoresistance by exploiting synthetic lethality in tumors with homologous recombination defects. Although BRCA mutations are rare in GBM, alterations conferring a “BRCAness” phenotype, including EGFRvIII, PTEN loss, and other DNA repair defects [205] and potentially, MGMT promoter methylation [206], may sensitize tumors to PARP inhibition and enhance responses to radiotherapy and chemotherapy.

Overall, clinical trials have so far not demonstrated a robust benefit of combining PARP inhibitors with TMZ [207,208,209], with the exception of a recent phase I study reporting encouraging activity of olaparib in combination with TMZ and radiotherapy in inoperable GBM. PARP inhibitors show limited blood–brain barrier penetration and overlapping hematologic toxicity when combined with TMZ or radiotherapy, restricting effective dosing. GBM cells can evade PARP inhibition through metabolic reprogramming, protective autophagy, and alternative DNA repair pathways, resulting in modest and inconsistent clinical benefit in unselected patient populations [83,210]. Nevertheless, clinical trials remain ongoing (NCT06258018, NCT02152982), and it is evident that successful implementation of PARP inhibition therapy in GBM will require a personalized, biomarker-driven approach.

### 7.2. ROS Modulation

Inducing oxidative stress in GBM cells by overwhelming their upregulated antioxidant defenses or increasing intracellular ROS beyond survival thresholds represents a promising strategy to overcome therapeutic resistance and selectively trigger tumor cell death [211].

High-dose ascorbate (HDA) is a promising pro-oxidant anticancer agent that generates intracellular H_2_O_2_ upon parenteral administration, inducing DNA damage and selective death of cancer cells with dysregulated iron metabolism, such as non-small cell lung cancer and GBM, while enhancing sensitivity to radio- and chemotherapy [212,213]. Clinical studies in newly diagnosed GBM have shown that HDA combined with TMZ and radiotherapy improves overall survival [214,215,216], and alterations in iron metabolism are being explored as potential biomarkers of therapeutic response [217] (NCT02344355). Arsenic trioxide induces ROS by inhibiting antioxidant defenses [113,218] and, when combined with radiotherapy and TMZ, has demonstrated acceptable toxicity and improved survival in specific GBM patient subgroups [219,220,221,222] (NCT00045565). In addition, preclinical studies show that targeting the glutathione-dependent antioxidant system in GBM using agents such as L-buthionine-S-sulfoximine, sulfasalazine, erastin, RSL3, or iron-modulating compounds induces ferroptosis and enhances sensitivity to chemotherapy [116,223,224,225]. However, clinical translation has been limited: sulfasalazine trials were terminated due to toxicity [223,226], while thioredoxin system inhibition with auranofin has shown cytotoxicity in vitro [227] and is currently under clinical evaluation within the CUSP9 protocol [228] (NCT02770378). In addition, the effect of redox therapy is limited due to profound GBM oxidative stress resistance [229]. Tumor regional heterogeneity in redox state and iron availability further complicates therapeutic efficacy, while systemic toxicity remains a concern for pro-oxidant approaches.

### 7.3. Autophagy Modulation

Targeting autophagy represents a promising strategy to sensitize GBM cells to therapy, either by inhibiting autophagic flux or by excessive activation leading to autophagy-mediated cell death [230]. The autophagy inhibitor chloroquine enhances TMZ efficacy by disrupting proteostasis, inducing endoplasmic reticulum stress, and promoting apoptosis [231], and its addition to standard therapy has prolonged survival in EGFRvIII-positive GBM patients [232]. Other clinical trials of chloroquine (NCT00224978) and hydroxychloroquine combined with conventional GBM treatment suggest therapeutic benefit (NCT00486603), though optimized dosing is still needed [233,234]. In parallel, preclinical studies show that pharmacological activation of autophagy using PI3K or mTORC/AKT inhibitors can suppress malignant GBM traits and enhance sensitivity to TMZ and radiotherapy [235,236].

However, autophagy modulation could be challenging in GBM due to its context-dependent dual role in promoting either cell survival under TMZ, hypoxia, and metabolic stress or cell death when excessively activated. This functional ambiguity complicates the definition of optimal timing, intensity, and direction of intervention, while clinical application is further limited by poor inhibitor specificity, uncertain dosing, and variable efficacy across molecular subtypes [237,238].

### 7.4. Targeting Metabolism

Another key feature of GBM that underlies its resistance to therapy is the high activity of diverse metabolic pathways, together with a high capacity for metabolic reprogramming.

Certain GBM subtypes and glioma stem cells depend on oxidative phosphorylation, making mitochondrial metabolism an attractive therapeutic target. Metformin inhibits mitochondrial complex I, disrupts ATP production [239], and has been shown in multiple preclinical studies to sensitize GBM to TMZ [239,240,241,242]. A recent meta-analysis reported improved overall survival with TMZ–metformin combination therapy compared with standard treatment, though optimal dosing requires further investigation [243].

At the same time, some GBM subtypes display enhanced aerobic glycolysis (the Warburg effect), characterized by upregulation of key glycolytic enzymes and transporters [244], creating a strong dependence on glucose metabolism to support tumor growth and immune evasion. This metabolic vulnerability has prompted investigation of glycolysis inhibition as a therapeutic strategy. Dichloroacetate shifts metabolism toward mitochondrial glucose oxidation by activating pyruvate dehydrogenase and has shown survival benefits in reported GBM cases, though clinical trials remain incomplete [245]. The glycolytic inhibitor 2-deoxy-D-glucose induces energy stress and exhibits cytotoxic and chemosensitizing effects in preclinical GBM models [246,247]. A major limitation of anti-glycolytic therapy is the systemic toxicity of glycolysis inhibitors necessitating careful dose titration and the development of selective delivery strategies.

Targeting mitochondrial dysfunction and lipid metabolism has emerged as a promising therapeutic strategy in GBM [248], as tumor cells rely on lipid accumulation and upregulated fatty acid and sterol biosynthesis to support growth and stress adaptation [249,250]. Fatty acid synthase (FASN), a key enzyme driving lipid anabolism, is frequently overactive in GBM and associated with poor prognosis [251]. Clinical evaluation of the FASN inhibitor denifanstat combined with bevacizumab has demonstrated significant survival benefits [252], underscoring the therapeutic potential of lipid metabolism–targeted approaches (NCT05118776).

Statins represent a potential therapeutic option in GBM by inhibiting HMG-CoA reductase and disrupting the mevalonate pathway required for tumor sterol biosynthesis and oncogenic signaling [253,254,255]. Preclinical studies show that statins exert antitumor effects and enhance the cytotoxicity of TMZ [256] and metformin [257], although clinical trials have demonstrated limited efficacy as monotherapy [258,259]. Notably, combination approaches integrating statins with dopamine receptor antagonists and radiotherapy have produced strong antitumor responses in vivo, highlighting their potential to improve GBM treatment outcomes [260].

Approaches targeting tumor metabolism are similarly constrained by profound metabolic plasticity. GBM cells can dynamically switch between glycolysis and oxidative phosphorylation, rewire lipid and iron metabolism, and exploit nutrient scavenging pathways to maintain energy homeostasis [244]. Inhibition of a single metabolic node often triggers compensatory adaptations and, in some cases, paradoxically enhances DNA repair capacity and therapy resistance [261]. Coexistence of metabolically distinct cell populations within individual tumors further undermines the efficacy of monotherapies and necessitates rational combination strategies [244]. Metabolic heterogeneity, and tumor plasticity undermine monotherapy efficacy [262], highlighting the need for carefully designed combination approaches [263,264].

### 7.5. Overcoming GBM Plasticity

Multiple therapeutic strategies have been explored to target GBM plasticity, although none have yet achieved durable clinical benefit. Epigenetic therapies have been extensively investigated as a means to constrain GBM plasticity by limiting transcriptional adaptability. Histone deacetylase (HDAC) inhibitors, BET bromodomain inhibitors, and modulators of Polycomb repressive complex 2 (PRC2) have shown the ability to suppress stem-like and mesenchymal programs and to sensitize GBM cells to radiotherapy in preclinical studies [265,266,267].

In GBM, aberrant expression of several HDAC family members contributes to epigenetic dysregulation, stemness, therapy resistance, and plasticity-associated transcriptional programs. HDAC inhibitors promote chromatin relaxation, impair DNA repair, activate tumor-suppressive pathways, and induce apoptosis or differentiation arrest in cancer stem cells [251,252]. In preclinical GBM models, HDAC inhibitors such as panobinostat, vorinostat, and romidepsin reduce tumor cell proliferation and invasion and enhance sensitivity to radiotherapy and chemotherapy, although limited blood–brain barrier penetration restricts clinical efficacy [253,254,255]. Clinical trials of vorinostat demonstrated acceptable tolerability and pharmacodynamic activity but only modest survival benefit, with greater sensitivity observed in proneural GBM [268]. In addition, a combination of vorinostat with TMZ is being currently tested (NCT00268385). Retrospective analyses have suggested potential survival benefits of valproic acid in select GBM subgroups, although results remain inconsistent and unconfirmed in prospective trials [256,257,258].

Overall, the clinical impact of HDAC inhibitors is limited by poor brain penetration, broad and non-selective transcriptional effects, and activation of compensatory survival pathways. Subtype-dependent responses highlight the need for molecular stratification and improved patient selection [269]. The complexity and heterogeneity of epigenetic landscapes across GBM tumors hinder the efficacy of HDAC inhibitors. Advances in epigenomic profiling may enable identification of tumors most susceptible to HDAC inhibition and support rational combination strategies aimed at constraining GBM plasticity and adaptive resistance [270].

Another major focus was on therapy-induced mesenchymal transition, a well-described adaptive response to radiation and chemotherapy. This transition is driven by stress-responsive signaling pathways, including STAT3, NF-κB, and TGF-β, which promote invasive behavior, immune evasion, and resistance [125,271]. Pharmacological inhibition of these pathways reduced mesenchymal features and tumor aggressiveness in experimental models, yet clinical translation has been hampered by pathway redundancy and context-dependent activation.

### 7.6. Targeting Glioblastoma Stem Cells

Direct targeting of GSCs represents a promising therapeutic strategy, as their stem-like properties are maintained by multiple signaling pathways [259,260,261], key transcriptional regulators [262,263], epigenetic regulators [264], and specialized microenvironments. Modulation of these mechanisms can promote GSC differentiation, thereby reducing tumor-initiating capacity and increasing sensitivity to chemotherapy and radiotherapy. In preclinical models suppression of key regulators such as SOX2, OLIG2, and FOXG1 [265] or inhibition of pathways including Notch, Wnt, Hedgehog, STAT3, and TGF-β enhances therapeutic responses, particularly in combination with standard treatments [260].

Despite these encouraging results, clinical evidence remains limited, with only early-phase trials completed and no robust survival benefit demonstrated to date [136]. For instance, activation of BMP signaling induces GSC differentiation and reduces tumorigenicity [266,267], and focal administration of recombinant BMP4 has shown safety and encouraging activity in a small clinical study [268]. Retinoids similarly promote GSC differentiation and, although they do not improve overall efficacy when added to standard therapy, have been associated with prolonged relapse-free survival when used as maintenance treatment [269,270]. Nevertheless, high redundancy among stemness-associated signaling pathways (Notch, Wnt, STAT3, TGF-β) allows rapid adaptation to targeted inhibition, while phenotypic plasticity enables escape from differentiation-based therapies [272]. Moreover, GSC populations differ among patients in terms of signaling pathways (e.g., Notch, Wnt, STAT3), microenvironmental niches, and metabolic states. Personalized approaches using patient-derived models and single-cell analyses can define the dominant GSC subpopulations and vulnerabilities in each tumor, enabling pathway-specific targeting and combination strategies (e.g., stemness blockade, immune modulation) more likely to impact survival [273].

### 7.7. Targeting Heterogeneity: Combination Strategies and Multitarget Drugs

Metabolic heterogeneity, and tumor plasticity undermine monotherapy efficacy [262], highlighting the need for carefully designed combination approaches. Combination strategies, mentioned in Section 2.2, and multitarget agents offer a potential strategy to overcome therapeutic resistance by simultaneously inhibiting multiple oncogenic signaling pathways, thereby reducing adaptive escape mechanisms. Multikinase inhibitors, which target several receptor tyrosine kinases, suppress tumor angiogenesis, invasion, and proliferation. However, clinical trials of pazopanib, sunitinib, and sorafenib in GBM have failed to demonstrate overall survival benefit, despite radiographic responses in selected patients [274,275,276]. In contrast, the multikinase inhibitor regorafenib has shown a statistically significant improvement in overall survival in recurrent GBM compared with standard therapy, supporting its clinical potential, although further validation in larger, molecularly stratified cohorts is required [277,278]. Regorafenib is currently being evaluated in an ongoing Phase II/III trial GBM AGILE (NCT03970447).

### 7.8. Conclusions and Future Perspectives

Despite extensive preclinical and clinical efforts, most strategies aimed at overcoming chemoresistance in GBM have not entered routine clinical practice due to limited efficacy and insufficient validation. The modest success of current approaches reflects not only inadequate potency of individual agents, but also adaptive resistance, pathway redundancy, intratumoral heterogeneity, and poor patient stratification. Progress will therefore require biomarker-guided combination therapies, improved drug delivery across the blood–brain barrier, and treatment designs that account for the dynamic evolution of GBM under therapeutic pressure.

The most clinically promising therapeutic directions in glioblastoma focus on mechanisms that can be exploited to overcome resistance and improve long-term outcomes. First, ferroptosis andHDA–induced oxidative stress represent a unified strategy to selectively kill glioma cells, including glioma stem cells, bypass DNA repair–mediated resistance, and synergize with chemotherapy, radiotherapy, and immunotherapy. Second, immune microenvironment reprogramming, including checkpoint inhibitors, CAR-T cells, vaccines, and oncolytic viruses, aims to overcome profound immunosuppression and induce durable antitumor T-cell responses [279]. Third, glioma stem cell targeting, via inhibition of Notch, STAT3, Wnt, or metabolic pathways, seeks to eradicate the subpopulation responsible for recurrence and chemoresistance. Fourth, modulation of epigenetic and transcriptional plasticity, through HDAC, BET, and DNMT inhibitors or blockade of mesenchymal transitions, limits adaptive phenotypic switching and resensitizes tumors to other therapies [280]. Finally, precision medicine and advanced delivery technologies, including biomarker-guided targeted therapies, and nanoparticle platforms, improve drug delivery and exploit specific molecular vulnerabilities.

Overall, chemoresistance in GBM is not governed by a single dominant mechanism but rather arises from a network of adaptive, patient-specific processes. Personalized therapeutic strategies that integrate molecular, functional, and cellular information offer a promising framework to address this complexity. Although challenges remain, advances in multiomics profiling, functional precision oncology, and patient-derived models support the rationale for moving beyond uniform treatment paradigms. Future biomarker-driven and adaptive clinical trials will be essential to determine whether precision medicine can significantly improve outcomes for patients with GBM.

## 8. Prospects for Personalized Approaches in GBM Therapy

Clinical trials of novel anticancer agents frequently demonstrate only modest improvements over standard-of-care therapies, largely because uniform treatment protocols fail to account for patient heterogeneity within the same histological diagnosis. Consequently, meaningful clinical benefit is often confined to small patient subsets, underscoring the impact of interpatient genetic, epigenetic, and therapeutic response variability and the need for personalized treatment strategies [281].

Despite extensive evidence of GBM heterogeneity, no standardized guidelines for personalized therapy selection have been established. Currently, MGMT promoter methylation is the only molecular biomarker routinely used in GBM prognostication; however, its role in treatment stratification remains limited. Clinical data indicate that patients with unmethylated MGMT promoters can still derive overall survival benefit from TMZ comparable to that of patients with methylated promoters relative to no TMZ treatment [7,282]. MGMT status has primarily influenced therapeutic decision-making in elderly patients, for whom TMZ monotherapy is preferentially administered to those with methylated promoters, whereas radiotherapy alone is recommended for patients with unmethylated promoters based on earlier evidence [283]. Other protein expression markers—including Ki-67 proliferation index, TP53 status, EGFR, ATRX, and GFAP—have been correlated with in vitro chemotherapy sensitivity and patient survival, suggesting potential predictive value when combined with drug response assays [284]. Emerging research also implicates DNA repair-related enzymes such as N-methylpurine-DNA glycosylase and STAT3 pathway activity as contributory mediators of chemoresistance and potential biomarkers for sensitivity modulation [285].

### 8.1. Genome-Wide Molecular Profiling

**Figure 2:**
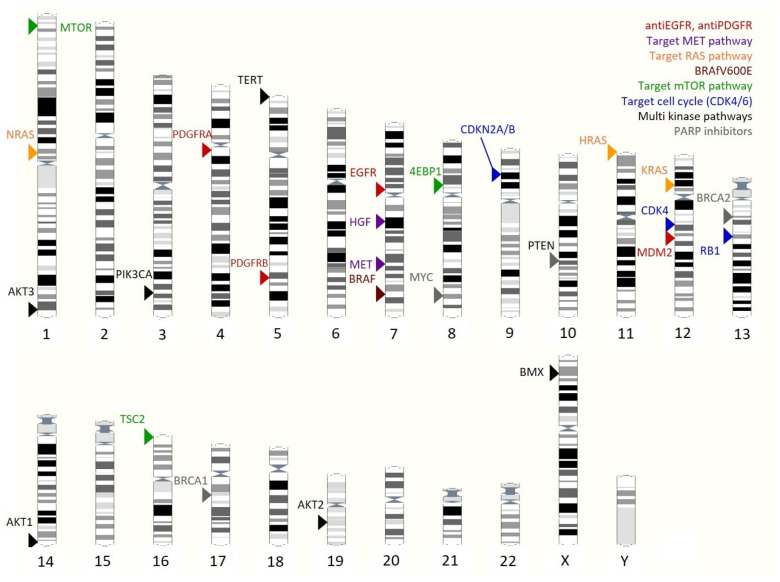
Frequently mutated oncogenes in the human genome in GBM with therapeutic potential, along with their chromosomal locations. 1–22, X, and Y indicate chromosome numbers; in the top right corner the main groups of targeted drugs are shown, and on the chromosomes the target genes for these drugs are highlighted in corresponding colors.

Genome-wide molecular profiling represents a key strategy for personalized therapy selection in solid tumors by enabling identification of targetable oncogenic driver alterations, and has been shown to improve response rates and survival across multiple cancer types [286]. In GBM, treatment regimens matched to tumor-specific molecular and genetic features demonstrate superior efficacy compared with empirically selected therapies [286,287]. As predictive molecular markers have been identified for many targeted agents, precision therapy guided by tumor molecular profiles represents a promising approach to improving treatment outcomes in GBM. The major biomarker genes for GBM targeted therapies are summarized and annotated by chromosomal location in Figure 2. Molecular stratification of patients with recurrent GBM using biomarkers such as PD-L1 expression, cyclin D1, mTOR activation, TERT mutation, CDKN2A/B loss, and BRAF V600E mutation enabled deployment of targeted agents and significantly improved overall survival compared with non-stratified treatment (13.0 vs. 4.3 months) [286].

Similar approaches in newly diagnosed GBM evaluated alterations in the p53, Hedgehog, and mTOR pathways, as well as ALK rearrangements. Patients with actionable alterations received matched targeted therapies, whereas others were treated with standard TMZ or alternative agents, including immunotherapy. Among targeted treatments, the mTOR inhibitor temsirolimus showed the greatest benefit in tumors with activated mTOR signaling, achieving a median survival of 15.4 months, while clinical efficacy of other agents remains unproven [288].

Nevertheless, identification of individual targetable molecular markers of a tumor is not always possible, and also biomarker presence does not reliably predict therapeutic response. Conversely, clinical benefit may occur in the absence of identifiable predictive markers, underscoring current limitations of biomarker-driven precision oncology in GBM [287,289,290,291].

### 8.2. Integrated Analysis of Complex Biomarkers

An integrative analysis of molecular biomarkers associated with prognosis and chemotherapeutic response represents a promising strategy for precision oncology. Numerous in silico approaches have been developed to identify clinically relevant molecular signatures across tumor types. For example, Decipher^®^ (Decipher Biosciences (Veracyte, Inc.), San Diego, CA, USA) and Oncotype DX^®^ (Exact Sciences Corp., Madison, WI, USA) are used to guide treatment decisions in prostate cancer [292,293]; Oncotype DX^®^, MammaPrint^®^ (Agendia BV, Amsterdam, The Netherlands), and PAM50 (Prosigna™, NanoString Technologies, Inc., Seattle, WA, USA) inform therapy selection in breast cancer [294,295]; a 23-gene signature supports prognostic stratification in lung cancer [296]; and the ClearCode34 classifier aids treatment selection in renal cell carcinoma [297].

In GBM, several gene expression signatures associated with TMZ resistance, comprising 8, 6, or 3 genes, have been reported [296,298,299]. Commercial platforms such as Foundation Medicine^®^ (Foundation Medicine, Inc., Boston, MA, USA), BostonGene^®^ (BostonGene, Waltham, MA, USA), Caris Life Sciences^®^ (Caris Life Sciences, Irving, TX, USA) and Tempus^®^ (Tempus Labs/Tempus AI, Inc., Chicago, IL, USA) provide comprehensive genomic and transcriptomic profiling using whole-exome and RNA sequencing. In addition, Epignostix^®^, (Heidelberg Epignostix GmbH, Heidelberg, Germany) based on the Heidelberg CNS Classifier [300], offers an open-access, genome-wide DNA methylation–based classification system that stratifies GBM into more than 100 epigenetic subtypes, enabling more precise molecular diagnosis. Despite their promise, routine clinical implementation of these platforms remains limited due to insufficient validation and a lack of robust evidence-based prognostic studies.

### 8.3. Functional Drug Sensitivity Testing Approaches

Functional drug sensitivity testing (DST) using patient-derived tumor cells or tissue fragments overcomes key limitations of molecular profiling approaches by enabling identification of effective therapies even in the absence of actionable driver mutations [290,301].

These approaches have been validated in hematological malignancies [302] and in a range of solid tumors, including ovarian, breast, colorectal, and head and neck cancers [303]. DST-guided therapy selection has demonstrated superior clinical efficacy and improved survival [291,304,305]. Reported sensitivities for predicting patient response range from 44.4% to 100%, with an average of approximately 98% [303].

For DST-guided personalized treatment selection, dissociated tumor cells, primary cultures, or intact tumor fragments can be used and maintained ex vivo or engrafted into immunodeficient animals [306]. Although culturing intact fragments preserves the native tumor microenvironment, this method is labor-intensive and poorly standardized. Murine patient-derived xenograft models closely recapitulate key biological features of the original patient tumors; however, their establishment requires prolonged timeframes, limiting their utility for time-sensitive, clinically actionable testing [307]. In contrast, assays based on dissociated cells or primary cultures from surgical samples are technically simpler, more reproducible, and more amenable to clinical implementation.

### 8.4. Culturing Strategies for DST

DST can be performed using freshly isolated post-surgical GBM cells [291,304] or cells expanded in vitro [305,308]. Importantly, culture conditions must preserve the molecular and cellular properties of the original tumor. Culturing step is important to minimize false-positive results due to isolation-induced cell death and to increase viable cell numbers for analysis.

The preservation of GSCs is a critical determinant of the translational relevance of patient-derived models, as these cells are implicated in tumor initiation, therapeutic resistance, and recurrence. Serum-free GBM cultures propagated in defined media supplemented with epidermal growth factor (EGF) and basic fibroblast growth factor (bFGF) selectively support cells with stem-like properties, including long-term self-renewal, multipotency, and expression of canonical GSC markers such as SOX2, OLIG2, CD133, and Nestin [309]. Importantly, these cultures retain tumor-initiating capacity upon orthotopic transplantation and recapitulate patient-specific genetic and epigenetic landscapes, indicating preservation of functionally relevant stem-like subclones. Serum-free GBM stem-like cell culture systems also maintain tumor genotype, gene expression profiles and clinically relevant chemoresistance patterns, including resistance to TMZ [167,309,310]. In contrast, long-term adherent cultures grown in the presence of serum induce astrocytic differentiation, transcriptional drift, and loss of tumorigenic capacity, leading to progressive depletion of the GSC compartment. Serum exposure has been shown to irreversibly alter gene expression profiles and attenuate tumor-initiating potential [311], rendering such models suboptimal for studying therapy resistance and relapse-driving populations in GBM. In additional studies, stem-like cell enrichment was achieved by culturing tumor cells in rotational bioreactors that simulate hypogravity conditions [305,312].

GSCs can be cultured under either two-dimensional (2D) or three-dimensional (3D) conditions. Compared with 2D monolayers, 3D systems such as neurospheres better mimic niche-associated features, maintaining stemness, quiescence, and functional heterogeneity, including proliferative and slow-cycling GSC states [181,313,314]. Sphere-based gradients activate hypoxia- and stress-related pathways, contributing to therapy resistance.

However, 3D cultures also have limitations: selection for anchorage-independent cells can alter clonal composition, hypoxic cores may induce non-physiological stress or necrosis, variability in size reduces reproducibility, and high-throughput screening is more challenging. Despite this, 3D models provide a physiologically relevant platform when combined with 2D approaches.

Two-dimensional serum-free cultures remain valuable for their simplicity, reproducibility, and compatibility with high-throughput drug testing [315,316,317,318]. Uniform exposure to nutrients and drugs enables robust quantitative readouts, making 2D systems informative for early drug discovery and hypothesis generation, especially when complemented by 3D models that preserve GSC diversity and niche-dependent phenotypes [315,316].

Beyond conventional 2D and 3D cultures, several more complex patient-derived models are being explored for improved clinical translatability in GBM drug testing [319,320]. Among these, patient-derived organoids are self-organizing three-dimensional structures that recapitulate key architectural, cellular, and molecular features of the parental tumor. GBM organoids demonstrate strong genotypic and phenotypic concordance with original tumors, preserving histology, cellular diversity, gene expression, mutational profiles, and morphological features, including nuclear atypia and limited vascular structures [321,322]. Their 3D organization enables more physiologically relevant cell–cell interactions compared with conventional cultures. However, GBM organoid generation may require up to 4 weeks and is technically complex, with lower success rates reported for IDH-mutant tumors compared with IDH-wild-type GBM [323]. A major limitation is a high risk of introduction of sampling bias due to inter-organoid heterogeneity derived from a single tumor that complicates DST [324,325]. Consequently, while patient-derived organoids offer enhanced physiological relevance for DST, challenges related to culture time and reproducibility remain.

Patient-derived tumor explants represent another ex vivo approach that preserves native tumor architecture and histology. Although explants have accurately predicted drug resistance in over 85% of non-CNS tumors, their application in GBM has been limited by low establishment rates, technical complexity, and strong dependence on tissue quality [326,327]. Moreover, each explant originates from a distinct tumor region, further complicating interpretation in the context of GBM heterogeneity.

Organotypic brain slice cultures constitute an additional emerging model in which patient-derived GBM tissue is implanted onto rodent brain slices, providing an intact tissue microenvironment [328]. These models demonstrate near-complete establishment rates and enable DST within approximately 8 days, aligning with clinically relevant decision timelines. However, their broader application is limited by the small number of available models, incomplete clinical validation, and the lack of an adaptive immune component in rodent-derived microenvironments. Recent studies using human brain slices from epilepsy surgery donors show promise [329], but further standardization and validation are required before routine implementation in DST.

### 8.5. Preclinical and Clinical Trials of Drug Sensitivity Profiling in GBM

**Figure 3:**
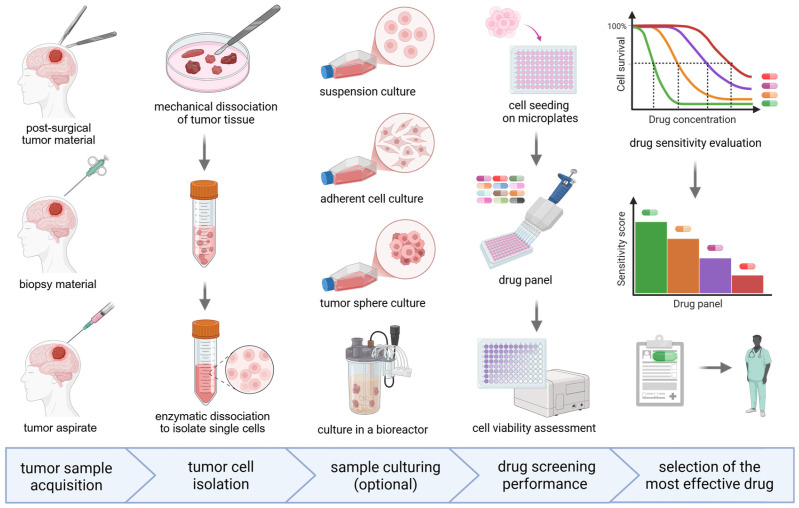
A summary of methods applied for DST of patient-tumor derived material for the best therapy selection. The process consists of several steps. Firstly, the tumor sample (parts of resected tumor, tumor biopsy or a tumor aspirate) is collected, and individual cells are dissociated from the tumor material using various mechanical and enzymatic methods. Isolated tumor cells may be cultured before DST performance or used immediately for the analysis. Based on the results of DST the most appropriate anticancer compound is selected and treatment recommendations are transferred to the clinicians. (figure created with BioRender.com/5zxjkew, accessed on 23 November 2025).

In GBM, multiple strategies using patient-derived cells and cultures have been developed for therapy selection. In vitro studies report high success rates (70–100%) in establishing GBM cultures and marked interpatient heterogeneity in drug sensitivity profiles, reflecting intertumoral variability [330,331,332]. It was shown that drug responses measured ex vivo correlate with patient survival, molecular-genetic tumor features, and clinical outcomes [291,333,334,335,336] (Table 1). Both 2D and 3D GSC culture systems have been validated for functional DST in GBM using collections of patient-derived tumor cultures [331,332]. Together, these findings support DST in GSC cultures as a robust and clinically relevant strategy for therapy selection in GBM patients. Key methodologies for tumor sampling, primary cell isolation and culture, drug screening, and candidate drug prioritization are summarized in Figure 3.

At least six prospective studies have investigated DST-guided therapy using patient-derived samples, including case series and clinical trials [291,304,305,338,339,340] (Table 2). Across these studies, patients who received treatments selected by ex vivo DST generally showed improved clinical outcomes, including overall survival and progression-free survival. However, most of these investigations were single-arm studies with small, heterogeneous patient cohorts, providing primarily evidence of feasibility and preliminary efficacy rather than definitive clinical benefit. Only one study was a randomized two-arm clinical trial [305] (NCT03632135). While this trial provided important early evidence that functional profiling can influence clinical outcomes in recurrent GBM, its impact is limited by the small sample size, heterogeneity in prior treatments, and its focus on the recurrent rather than first-line setting. An ongoing phase II trial, ATTRACT (NCT06512311) addresses these challenges by enrolling a larger cohort of newly diagnosed GBM patients within a defined molecular subgroup (IDH-wildtype, MGMT-unmethylated) [341]. Taken together, although ex vivo DST shows feasibility and potential clinical value for GBM therapy selection, its benefit must be confirmed in large, randomized, well-controlled prospective trials before clinical adoption.

## 9. Limitations of Functional Drug Sensitivity Testing

### 9.1. Preserving GBM Heterogeneity

A major limitation of functional precision oncology in GBM is that drug response profiles strongly depend on the cell culturing strategy. Freshly isolated post-surgical cells preserve native tumor composition, but are limited by low numbers and viability. Short-term serum-free cultures allow limited expansion while partially retaining heterogeneity, yet even brief in vitro propagation can alter clonal representation and cellular states [309,342]. Because GBM recurrence is driven by pre-existing resistant subclones [160,343,344], their loss during culture limits the predictive value of DST. Overall, the culturing step acts as a biological filter, and translational relevance is maximized in systems that minimize culture-induced selection while remaining experimentally tractable.

Nevertheless, early-passage primary cultures, GSC models, and three-dimensional organoids retain key molecular and transcriptional features of the original tumor, including coexisting subclones. This allows simultaneous assessment of drug effects across multiple clinically relevant cellular states [345]. DST integrates determinants of therapy response, such as DNA damage repair capacity, epigenetic programs, signaling pathway redundancy, and stress adaptation [346]. Consequently, DST profiles derived from patient-specific cultures have shown meaningful correlations with clinical responses in GBM [335,336].

### 9.2. Addressing Practical Limitations

Despite its potential, several practical and economic constraints currently limit the widespread clinical implementation of patient-derived DST. Personalized drug selection is resource-intensive: tumor sample processing, establishment and maintenance of primary cultures or organoids, specialized laboratory infrastructure, and labor-intensive DST protocols contribute to high costs. These expenses increase further when testing multiple compounds or drug combinations. Moreover, such approaches are not routinely reimbursed by insurance systems, and although their clinical benefit appears promising, it has not yet been universally validated, limiting cost-effectiveness and scalability [347,348].

Scalability represents another major challenge. While high-throughput screening with established cancer cell lines has facilitated pharmacological profiling for drug discovery, adapting these platforms to individualized patient cultures is considerably more complex. Primary tumor cultures often grow at variable rates and require tailored protocols for isolation and expansion, complicating standardization and automation [347]. Additionally, the lack of universally optimized culture conditions and reproducible functional readouts across laboratories restricts throughput and comparability. Developing scalable workflows that integrate reliable cell expansion, robust assay endpoints, and automation is therefore essential for cost-effective, widely deployable personalized DST in oncology [347].

Limited availability and quality of surgical tumor specimens contributes to the practical constraints for DST in GBM. Only small tissue samples can be safely obtained without compromising a patient’s neurological function, and successful primary culture establishment requires rapid processing of fresh tissue to preserve both viability and heterogeneity. While viable cultures can often be generated within approximately one week, their success depends on tumor size, surgical accessibility, and tissue quality. Repeat surgical resection is not routinely performed in GBM and is typically limited to selected cases of recurrence, further restricting access to fresh tumor material. In some instances, stereotactic biopsy may be used to obtain tissue from inoperable tumors; however, this is not always feasible due to tumor location or clinical contraindications [349].

Time constraints present an additional translational limitation. GBM progresses rapidly, making turnaround time a critical factor for clinical utility. Ex vivo drug screening platforms in various cancers can generate predictive response data within 7–10 days after surgery, allowing integration into multidisciplinary treatment planning [350,351,352]. In contrast, patient-derived xenograft (PDX) models require 4–8 months for tumor engraftment and DST, rendering them impractical for individualized therapy in GBM [353]. Conventional GSC culture-based profiling may take up to 10 weeks, often exceeding clinically relevant timelines [331]. Parallelized GSC isolation strategies can reduce culture establishment to approximately three weeks with high success rates (>90%) [332], but these methods remain resource-intensive. Emerging rapid functional assays using 3D culture systems show promise in delivering reliable drug response data within ~7 days, aligning DST more closely with GBM clinical timelines [291]. Nevertheless, an optimized culture step remains essential to minimize isolation-induced artifacts and ensure a sufficient proportion of viable cells for robust drug testing.

In conclusion, therapy selection for GBM patients based on tumor cell cultures is theoretically feasible but practically limited due to high tumor heterogeneity, difficulties in cell cultivation, absence of the tumor microenvironment and immune system, long testing times, and lack of standardization. Currently, the method remains experimental, although it shows promising results in research settings.

## 10. Integrating Patient-Derived Cell Approaches with Molecular Testing in GBM

Integrating genomic profiling with phenotypic DST in patient-derived GBM cell cultures provides a powerful strategy to bridge the gap between tumor genotype and therapeutic vulnerability. Traditional sequencing approaches often fail to capture the full spectrum of tumor diversity, resulting in suboptimal therapeutic guidance. In this context, DST using patient-derived tumor cultures has emerged as a promising alternative, precisely because it preserves the heterogeneity of the tumor rather than attempting to simplify it. Therefore, functional profiling can sometimes predict optimal therapeutic regimens more accurately than NGS-based approaches, identifying patients who may benefit from targeted therapies despite lacking the canonical mutations [291].

MGMT promoter methylation is the most robust predictor of TMZ benefit; however, treatment response remains heterogeneous even among methylated tumors, while subsets of MGMT-unmethylated tumors retain partial sensitivity, indicating additional DNA damage response mechanisms [354,355]. These discrepancies highlight the limitations of genomic biomarkers alone. DST enables direct, patient-specific assessment of drug sensitivity and pathway dependence, often revealing discordance between molecular markers and therapeutic response [309]. TMZ resistance has been observed in MGMT-methylated patient-derived models, whereas partial sensitivity persists in some unmethylated tumors [334,335].

Similarly, EGFR amplification or mutation in GBM does not reliably predict response to EGFR-targeted therapies [356]. Integrating functional assays with genomic profiling can identify true EGFR-dependent tumors, refine patient selection, and uncover combinatorial vulnerabilities, thereby improving precision therapy strategies. Thus, integrating molecular diagnostics with patient-derived functional assays represents a promising strategy to refine therapeutic selection and advance personalized treatment approaches in GBM.

Notably, phenotypic testing as a standalone approach has important limitations. Drug responses are highly context-dependent and sensitive to experimental conditions, and without genomic or transcriptomic annotation, mechanistic interpretation of sensitivity or resistance remains limited [357]. Moreover, phenotypic assays may primarily reflect dominant clones at the time of sampling and fail to predict evolutionary escape driven by genetically defined resistant subpopulations [355]. Accordingly, in GBM, phenotypic DST is most informative when integrated with genomic profiling, which provides a stable framework for mechanistic understanding, biomarker development, and translational reproducibility.

## 11. Conclusions

Current therapeutic regimens for GBM remain largely ineffective, with relapse occurring in the majority of patients and only modest survival gains achieved with TMZ-based therapy. Although numerous cytotoxic and targeted agents have been evaluated, none has provided a clinically meaningful survival advantage beyond the current standard of care. A central obstacle to therapeutic success is the profound inter- and intratumoral heterogeneity of GBM, coupled with marked cellular plasticity, which collectively drive highly variable treatment responses and adaptive resistance.

Accumulating evidence indicates that GBM chemoresistance arises from a complex and interconnected network of intrinsic and adaptive mechanisms, including enhanced DNA repair capacity, apoptotic dysregulation, signaling redundancy, metabolic reprogramming, oxidative stress tolerance, autophagy activation, and drug efflux. These tumor-intrinsic factors are further reinforced by microenvironmental influences, immune suppression, and the blood–brain barrier, limiting the efficacy of conventional cytotoxic approaches. Importantly, resistance is frequently sustained by pre-existing resistant subclones and dynamic therapy-induced cell-state transitions, particularly within glioma stem cell (GSC) populations, underscoring the limitations of uniform treatment paradigms.

Emerging strategies, including ferroptosis induction, targeting of GSCs, nanotechnology-based drug delivery, tumor microenvironment modulation, immunotherapy, and rational combination regimens, offer promising avenues to disrupt adaptive resistance networks. However, monotherapies directed at individual resistance nodes have thus far yielded limited clinical benefit, largely due to pathway redundancy and compensatory signaling. These observations support a shift toward precision-guided, multimodal treatment strategies capable of addressing tumor complexity at multiple biological levels.

Functional precision oncology approaches, including drug sensitivity testing in patient-derived cultures, represent a biologically rational strategy to tailor therapy to the most resistant tumor compartments. In GBM, GSC-based platforms offer particular promise given their capacity to recapitulate patient-specific drug responses. Nevertheless, despite encouraging preclinical and early clinical findings, culture-based personalization has not yet been incorporated into clinical guidelines. Key barriers include limited prospective randomized evidence, lack of interlaboratory standardization, incomplete modeling of tumor–microenvironment interactions, high costs, and logistical and regulatory challenges.

Future progress will depend on improving the biological fidelity and scalability of ex vivo models, integrating functional assays with multi-omics profiling and computational analytics, and validating these approaches in well-designed prospective trials. Rather than functioning as standalone decision tools, culture-based assays are likely to become components of integrated precision oncology frameworks that combine molecular, functional, and clinical data to guide adaptive therapeutic strategies.

In conclusion, although no personalized strategy has yet transformed the standard of care for GBM, growing mechanistic insight into chemoresistance and advances in functional and molecular profiling provide a strong rationale for continued development of individualized, combination-based approaches. A deeper integration of biological understanding with clinically actionable testing platforms may ultimately translate into more durable disease control and meaningful survival benefits for patients with GBM.

## Figures and Tables

**Table 1:** Data from Preclinical Studies and Validation of Personalized Chemotherapy Selection Methodologies for GBM Using High-Throughput ex vivo Chemotherapeutic Drug Screening.

Reference	Patient Cohort	Sample Type	Success * Rate	Culture Medium	Culture/Sample Model	Drug Panel	Drug Screen Performance	Sensitivity Criteria	Heterogeneity Evidence	Clinical Correlation and Predictive Power
Skaga et al., 2019 [330]	12 ND GBM	Biopsy + UA	12/12 (100%)	Stem cell **	Tumorspheres + adherent	461 cmp	384-well; 3000 cells/well; 72 h exposure; CTG	DSS ≥ 10	High intertumoral drug-response heterogeneity	Not provided
Skaga et al., 2019 [331]	10 R GBM	Biopsy + UA	7/10 (70%)	Stem cell	Tumorspheres + adherent	525 cmp incl. TMZ	384-well; 3000 cells/well; 72 h exposure; CTG For TMZ: 96-well; 5000 cells/well; 10 d exposure; XTT	DSS ≥ 10	High intertumoral drug-response heterogeneity	Not provided
Skaga et al., 2022 [334]	51 ND/R GBM	Biopsy + UA	(51/51) 100%	Stem cell	Tumorspheres + adherent	TMZ	96-well; 5000 cells/well; 10 d exposure; XTT	DSS ≥ 10	Difference between ND and R GBM in TMZ sensitivity	DST-pr responders: OS 14 vs. 10.5 mo; MGMT-methylated subgroup had longer OS
Howard et al., 2017 [333]	41 ND GBM	Biopsy	(41/41) 100%	FBS- containing	CSCs: 3D in a bioreactor BTCs: adherent	13 cmp incl. TMZ + 2 comb.	96-well; 1000 cells/well; 1 h exposure; WST8 48 h later	CSC-test: ≥40% CSC kill; Bulk test: ≥55% BTC kill	Not provided	CSC test: DST-pr responders PFS 20 vs. 3 mo; Bulk test: DST-pr responders PFS 13 vs. 4 mo; PDX-validated effectiveness
Shuford et al., 2021 [291]	55 HGG, (incl. 29 ND GBM)	Resection/ biopsy	58/61 (95%)	FBS- containing	Spheroids	13 cmp incl. TMZ	384-well; 24 h spheroid formation; 72 h exposure; CTG3D	IC50 ≤ 25th quantile	Not provided	DST-pr accuracy was 85% for ND GBM DST-pr responders: OS 11.6 vs. to 5.9 mo
Ledford et al., 2024 [336]	59 ND HGG, (incl. 52 GBM)	Resection/ biopsy	86/99 (87%)	FBS- containing	Spheroids	TMZ	384-well; 24 h spheroid formation; 72 h exposure; CTG3D	IC50 quantiles	Not provided	DST-pr responders: OS 16.8 vs. 11.7 mo; PFS 9.1 vs. 4.4 mo; No correlation with MGMT status
Nam et al., 2023 [167]	69 ND GBM	Resection	Not provided	Stem cell	Suspension	TMZ	384-well; 500 cells/well; 6 d exposure; ATPL1	GR50 z-score ≤ 0; AUC z-score ≤ 0	High intertumoral molecular and drug-response heterogeneity	DST-pr responders: OS ~21 vs. ~10 mo; PFS ~13 vs. 5 mo;
Ntafoulis et al., 2023 [335]	66 ND GBM	Resection	66/66 (100%)	Stem cell	Adherent	TMZ	96-well; 1000 cells/well, 6 d exposure; CTG2.0	<50% viability at 100 μM TMZ	High intertumoral molecular heterogeneity	DST-pr responders: OS 27.8 vs. 15.9 vs. 12.3 mo; MGMT-methylated subgroup had better outcome
Verheul et al., 2025 [332]	31 ND/R GBM	Biopsy + UA	22/23 (96%)	Stem cell	Adherent	21 cmp incl. TMZ	384-well; 500 cells/well; 5 d exposure; CTG2.0	Not provided	High intertumoral morphologic, transcriptomic, drug-response heterogeneity	Not provided

Abbreviations: ND GBM—newly diagnosed GBM; R GBM—recurrent GBM; HGG—high grade glioma; UA—ultrasonic aspirate; CSCs—cancer stem cells; BTCs—bulk tumor cells; DST—drug sensitivity test; DST-pr—DST-predicted; DSS—drug sensitivity score [337]; CTG, CTG3D, CTG2.0 (CellTiter Glo (3D) (2.0)) and ATPL1 (ATPLite1step)—luminescent cell proliferation assays based on ATP quantification; XTT, WST8—colorimetric cell proliferation assays based on the reduction in corresponding tetrazolium salts; GR50—half inhibitory growth rate inhibitory concentration; AUC—area under curve; OS—median overall survival; PFS—median progression-free survival. * Success rate indicates the proportion of tumor samples that, after isolation, yield either a viable cell culture or a sample of sufficient quality for DST. ** Stem cell medium is a serum-free medium typically containing a supplement for culturing neuronal cells (B27 and N2) and growth factors (EGF and bFGF) to maintain growth and self-renewal of GSCs in vitro [309].

**Table 2:** Clinical trial data on personalized therapy selection methodologies for GBM using high-throughput ex vivo chemotherapeutic drug screening.

Reference	Clinical Trial ID and Name	Patient Cohort	Clinical Workflow	Culture Medium	Culture/Sample Model	Drug Panel	Drug Screen Performance	Drug Sensitivity Criteria	Clinical Outcome (DST-Guided)
Iwadate et al., 2003 [304]	N/A	31 ND GBM	Resection → DST → DST-pr therapy + RT or RT for DST-pr NR	FCS-containing	Single cell suspension	30 cmp	8 h exposure; FC 72 h later	≥20% reduction in G0/G1	OS 20.5 mo; No CR; 26% PR; 65% SD
Iwadate et al., 2010 [338]	N/A	74 ND GBM	Resection → DST → DST-pr therapy + RT or PCV for DST-pr NR	FCS-containing	Single cell suspension	25 cmp	8 h exposure; FC 72 h later	≥20% reduction in G0/G1	OS 19.4 mo PFS 9.2 mo
Ranjan et al., 2020 [339]	NCT03632135	14 HGG (12 R GBM)	Resection → DST → DST-pr therapy	FBS-containing	CSCs: 3D in a bioreactor BTCs: adherent	9 cmp + 5 comb.	96-well; 1000 cells/well; 1 h exposure; MTT 24 h later	≥40% CSC kill; ≥55% BTC kill	OS 13.3 (DST-treated) vs 9.0 mo (historical data); 43% CR; 43% PR; 14% PD
Ranjan et al., 2023 [340]	NCT03632135	40 R GBM	Biopsy → DST → DST-pr HR therapy (*n* = 29) or LR therapy (*n* = 11)	FBS-containing	CSCs: 3D in a bioreactor BTCs: adherent	9 cmp + 5 comb.	96-well; 1000 cells/well; 1 h exposure; WST8 48 h later	≥40% CSC kill; ≥55% BTC kill	OS 22.4 mo (DST-pr HR therapy) vs 12.5 mo (DST-pr LR therapy)
Ranjan et al., 2023 [305]	NCT03632135	78 R GBM	Resection/biopsy → DST → randomization → DST-pr therapy (*n* = 43) or physician choice (*n* = 35)	FBS-containing	CSCs: 3D in a bioreactor BTCs: adherent	9 cmp + 5 comb.	96-well; 1000 cells/well; 1 h exposure; WST8 48 h later	≥40% CSC kill; ≥55% BTC kill	OS 12.5 mo, PFS 6.5 mo (DST-guiding group) vs. OS 9 mo, PFS 3.3 mo (physician-choice group);
Schuford et al., 2021 [291]	NCT03561207	7 R HGG (6 GBM)	Resection/biopsy → DST → DST-pr therapy	FBS-containing	Spheroids	6 cmp incl. TMZ	384-well; 24 h spheroid formation; 72 h exposure; CTG3D	IC50 ≤ 25th quantile	PFS 7.9 mo
Berghoff et al., 2025 [341]	NCT06512311	240 ND GBM (planned)	Resection → randomization → RT + TMZ → DST → DST-pr therapy (*n* = 120) or no additional treatment (control group, *n* = 120)	Not mentioned	Not mentioned	28 cmp	384 well; 500 cells/well; 7 d exposure; CTG3D	Will be calculated based on AUC	Expected results: OS 17 mo (DST-guided group) vs 12 mo (control group)

Abbreviations: ND GBM—newly diagnosed GBM; R GBM—recurrent GBM; HGG—high grade glioma; DST—drug sensitivity test; DST-pr—DST-predicted; NR—nonresponders; RT—radiation therapy; PCV—a combination of procarbazine, lomustine and vincristine; FCS—fetal calf serum; FBS—fetal bovine serum; CSCs—cancer stem cells; BTCs—bulk tumor cells; FC—flow cytometry; CTG3D—CellTiter Glo 3D—a luminescent cell proliferation assay based on ATP quantification; MTT, WST8—colorimetric cell proliferation assays based on the reduction in corresponding tetrazolium salts; OS—median overall survival; PFS—median progression-free survival; HR therapy—high-response therapy; LR therapy—low response therapy; CR—complete response; PR—partial response; SD—stable disease; PD—progressive disease; N/A—not available; ‘→’ indicates the progression from one clinical step to another.

## Data Availability

This study is a literature review; all data are obtained from published sources. Further inquiries can be directed to the corresponding author.
